# Mathematical (Dis)abilities Within the Opportunity-Propensity Model: The Choice of Math Test Matters

**DOI:** 10.3389/fpsyg.2018.00667

**Published:** 2018-05-08

**Authors:** Elke Baten, Annemie Desoete

**Affiliations:** ^1^Department of Experimental Clinical and Health Psychology, Ghent University, Ghent, Belgium; ^2^Department of Speech and Language Pathology, University College Arteveldehogeschool, Ghent, Belgium

**Keywords:** Opportunity-Propensity Model, Mathematical Learning Disabilities, temperament, personality, motivation, subjective well-being, self-esteem, self-perceived competence

## Abstract

This study examined individual differences in mathematics learning by combining antecedent (A), opportunity (O), and propensity (P) indicators within the Opportunity-Propensity Model. Although there is already some evidence for this model based on secondary datasets, there currently is no primary data available that simultaneously takes into account A, O, and P factors in children with and without Mathematical Learning Disabilities (MLD). Therefore, the mathematical abilities of 114 school-aged children (grade 3 till 6) with and without MLD were analyzed and combined with information retrieved from standardized tests and questionnaires. Results indicated significant differences in personality, motivation, temperament, subjective well-being, self-esteem, self-perceived competence, and parental aspirations when comparing children with and without MLD. In addition, A, O, and P factors were found to underlie mathematical abilities and disabilities. For the A factors, parental aspirations explained about half of the variance in fact retrieval speed in children without MLD, and SES was especially involved in the prediction of procedural accuracy in general. Teachers’ experience contributed as O factor and explained about 6% of the variance in mathematical abilities. P indicators explained between 52 and 69% of the variance, with especially intelligence as overall significant predictor. Indirect effects pointed towards the interrelatedness of the predictors and the value of including A, O, and P indicators in a comprehensive model. The role parental aspirations played in fact retrieval speed was partially mediated through the self-perceived competence of the children, whereas the effect of SES on procedural accuracy was partially mediated through intelligence in children of both groups and through working memory capacity in children with MLD. Moreover, in line with the componential structure of mathematics, our findings were dependent on the math task used. Different A, O, and P indicators seemed to be important for fact retrieval speed compared to procedural accuracy. Also, mathematical development type (MLD or typical development) mattered since some A, O, and P factors were predictive for MLD only and the other way around. Practical implications of these findings and recommendations for future research on MLD and on individual differences in mathematical abilities are provided.

## Introduction

Mathematical competence relies on several interrelated mechanisms and skills (Siemann and Petermann, 2018). Procedural skills are required to understand principles and solve calculations in a number problem (e.g., 48 + 6 = …) or in a word problem (e.g., 6 more than 48 is …) format. Additionally, mathematical competence relies on the capacity to remember and retrieve arithmetic facts (e.g., 16 : 4 = …) with ease. Therefore, mathematics is considered to be componential in nature (Dowker, 2015). Research shows a lot of individual variation in school-taught mathematical abilities from the first year of primary school onwards (Mooij and Driessen, 2008; Clements and Sarama, 2011; Schuchart et al., 2015). To provide insight into the nature of these differences, some studies focused on predictors for mathematical outcomes, whereas others have compared children with and without Mathematical Learning Disabilities (MLD). MLD is a neurodevelopmental disorder characterized by mathematic skills substantially lower than expected with regard to the individual’s chronological age and by persisting math problems despite interventions that target those difficulties (Bryant et al., 2015; Pieters et al., 2015; Baten et al., 2017). Worldwide, the prevalence of MLD is estimated between 5 and 7% (Shalev, 2007; Shin and Bryant, 2015). In addition, some authors propose that MLD is a heterogeneous disability with a procedural and a semantic memory subtype (Henik et al., 2015). The procedural subtype is characterized by a delay in the acquisition of procedural calculation procedures. In contrast, the semantic memory subtype is marked by a lack of fact retrieval fluency (Pieters et al., 2015).

Previous research focused on domain-specific cognitive predictors of mathematics, such as symbolic numerical processing (Vanbinst et al., 2015) and seriation and classification (Stock et al., 2010) in pre-school. In addition, studies demonstrated the relationship between domain-general cognitive abilities such as intelligence (Desoete, 2008; Dix and van der Meer, 2015) and working memory (De Weerdt et al., 2013) on the one hand, and mathematical abilities on the other hand. Moreover, socioeconomic status (SES; Jordan and Levine, 2009; Aunio and Niemivirta, 2010) and parental academic aspirations (Murayama et al., 2016) were studied as contextual predictors. Finally, some researchers focused on non-cognitive predictors such as personality (e.g., Poropat, 2009) and motivation (Ryan and Deci, 2000; Froiland and Worrell, 2016).

However, by focusing on single predictors, the importance and unique explained variance of these predictors could be overestimated. Surprisingly few studies have been conducted to explore the combined effect of predictors. This study addresses this gap by investigating multiple predictors at the same time, within a comprehensive model to get a more holistic insight on math development. In what follows, we describe the model that will be used.

**FIGURE 1:**
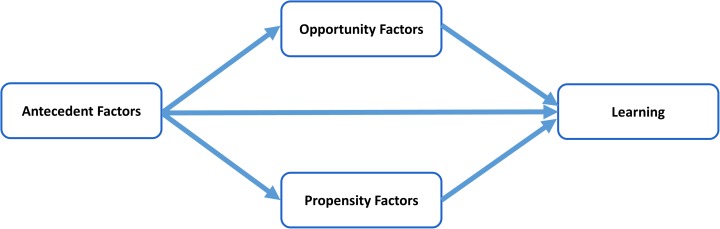
The Opportunity-Propensity Model. Adapted from Byrnes and Miller (2007, p. 602).

Byrnes and Miller (2007) developed the Opportunity-Propensity (O-P) framework, aiming to differentiate between opportunity (O) and propensity (P) factors in an effort to explain variance and individual differences in development. P factors are variables that make people able (e.g., intelligence) and/or willing (e.g., motivation) to learn. O factors include contexts and variables that expose children to learning content (e.g., home environment, classroom instruction). Antecedent (A) or distal variables, for example SES, are present early in a child’s life and explain why some people are exposed to richer O contexts and have stronger P’s for learning than others (Byrnes and Miller, 2007, 2016; Wang and Byrnes, 2013; Ceulemans et al., 2017). A visual representation of the model can be found in **Figure 1**.

The O-P Model has been tested by using secondary datasets. However, these studies are still scarce, since there are only three studies about this. In the first longitudinal study, researchers explained about 80% of variance through A, O, and P factors in secondary school children in the United States who were followed from 8th up until 10th grade. Path analysis confirmed the causality between A factors on the one hand and O and P factors on the other hand, as well as causality between the latter two and math achievement. Although the effect of A factors was mediated through O and P factors, A factors had a direct but small effect on math results (Byrnes and Miller, 2007). A second longitudinal study with data from kindergarten up until primary school revealed additional evidence for the O-P Model with P factors as the strongest predictors (Byrnes and Wasik, 2009). Finally, Wang et al. (2013) found evidence for this model in lower-income pre-kindergarten children. Using Structural Equation Modeling (SEM), it was confirmed that the latent A factors predicted both the latent O and P factors and the latent O factor predicted early math skills. The predictive value of the latent P factor was not confirmed. However, significant predictions for early math skills could be made based on intelligence and self-regulation as P factors.

Because the current study intends to combine variables in an O-P Model and because there are to the best of our knowledge only three studies combining different predictors (see previous paragraph), the results of research examining these variables separately will be summarized here. Furthermore, all variables will be categorized as A, O, or P variables.

Studies including A indicators, revealed the role of SES in math development, especially in low-income families (Wang et al., 2013). Moreover, parental stimulation has also been related to mathematical achievement, although it remains unclear whether this relation was direct or mediated through intelligence or the availability of certain resources such as books, computers, etc. (Blevins-Knabe et al., 2007; Kleemans et al., 2012; Niklas et al., 2016). In addition, lower birth weight was related to lower levels of math performance at school-age level, with especially strong effects for extremely low birth weight (<1500 g; De Rodrigues et al., 2006; Chatterji et al., 2014). Finally, children who are born first seem to perform better in academic contexts. This has been explained by the dilution hypothesis in which the first born child takes advantage of more parental resources (at least for the time the child is an only child), compared to later born children who have to share these resources (Hotz and Pantano, 2015).

Studies including P indicators demonstrated that motivation, personality, temperament, intelligence, and working-memory capacity as well as well-being variables, predicted mathematics. In a meta-analysis on 18 studies, Taylor et al. (2014) highlighted a positive relationship between autonomous motivation (where the force to fulfill a task is internal, e.g., passion) and general school achievement, in addition to a negative relationship between controlled motivation (where the force to fulfill a task is external, e.g., rewards-related) and academic achievement. According to research on the Big Five Personality Theory (Costa and McCrae, 1992), conscientiousness and openness are the personality traits most strongly associated with better academic performances, even when controlling for intelligence (Poropat, 2009; Zhang and Ziegler, 2016). Furthermore, math performance correlated positively with emotional stability (Zhang and Ziegler, 2016). Temperament, which is considered as the biological base of personality and described by the Reward Sensitivity Theory (Gray, 1981) as mechanisms guiding human behavior in terms of reactivity and self-regulation, can be seen as a P factor. More specifically, the unique constellation of one’s temperament could make people willing and able to learn (Van Beek et al., 2013). Research on 565 Dutch University students revealed that pursuing rewards or positive consequences (higher Behavioral Activation System – BAS) was associated with higher study engagement and better academic performances. A temperament characterized by trying to avoid punishment or negative consequences (higher Behavioral Inhibition System – BIS) was related to more overcommitment and lower academic performances through exhaustion (Van Beek et al., 2013). Studies on intelligence and working-memory showed positive correlations with mathematical abilities (Roth et al., 2015; Peng and Fuchs, 2016). Moreover, well-being can be considered a P factor, since it makes people willing and able to learn. Positive and bidirectional relations between subjective well-being (SWB) and academic performance were found. For instance, Quinn and Duckworth (2007) revealed in 257 fifth grade students that higher levels of SWB (indicated by high levels of life satisfaction as cognitive component; and more positive emotions than negative emotions as affective component) were related to better academic performance and vice versa. This relationship was significant even when controlling for intelligence. Furthermore, higher perceptions of own academic competence were predictive of better academic achievement and the other way around (Arefi et al., 2014) which confirmed the reciprocal-effects model between academic self-concept and academic achievement (Guay et al., 2003; Seaton et al., 2015).

As to the O factors, teaching methods (Savelsbergh et al., 2016), instructional time (Cattaneo et al., 2016), teacher education level, and teachers’ years of experience (Zhang, 2008) were found to be responsible for more O’s to learn. The impact of the O factors depended on the specific support factors (Byrnes and Wasik, 2009; Cowan, 2015).

### The Current Study

This study aimed to add some nuance to the literature on individual differences in mathematics learning by combining A, O, and P indicators within the O-P Model. Although there is already some evidence for this model (Byrnes and Miller, 2016, 2007; Byrnes and Wasik, 2009; Wang and Byrnes, 2013) from secondary datasets, there is little research from primary data simultaneously tapping the A’s, O’s, and P’s empirically in children with and without MLD. Therefore, this study had the objective to extend the literature on the O-P Model in several ways. First, a variety of non-cognitive variables that had not yet been investigated in the context of this theory (e.g., temperament, personality, and self-perceived competence) were included. Second, the current study investigated specificity and examined differences between children with and without MLD on A’s and P’s and explored if there were different relationships with outcome depending on group (MLD or control). As such, this study contributes to theory-building about mathematical learning since it investigates whether the same learning models can be applied for children with and without clinical diagnosis. Finally, this study expands previous findings by taking the componential nature of mathematics into account by separately examining the prediction for procedural calculation and fact retrieval skills among children (Cohen Kadosh and Dowker, 2015; Pieters et al., 2015).

**FIGURE 2:**
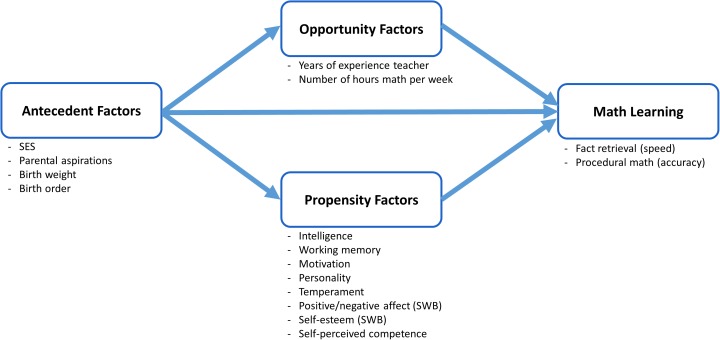
Operationalization of the O-P Model in this study. Adapted from Byrnes and Miller (2007, p. 602).

The operationalization of the O-P Model in the current study is described in **Figure 2**. Four major hypotheses were examined:

(1) There will be differences in A and P indicators between children with and without MLD. We expect children with and without MLD to differ on these specific variables in a way that the negative predictors (Hypotheses 2 and 3) for math performance will be higher in children with MLD and vice versa.
(2) A selection of A, O, and P indicators will predict fact retrieval speed.
(3) A selection of A, O, and P indicators will predict procedural accuracy.
(4) The predictive value of some A variables will be mediated through some O and P variables.

For Hypotheses 2 and 3, based on the literature described above, it is expected that children will have better mathematical abilities when they are raised with more O’s (Zhang, 2008; Byrnes and Wasik, 2009), higher levels of SES (Wang et al., 2013), higher parental aspirations (Blevins-Knabe et al., 2007; Kleemans et al., 2012; Niklas et al., 2016), were born with higher birth weight (De Rodrigues et al., 2006; Chatterji et al., 2014) and have a higher place in the birth order (Hotz and Pantano, 2015). Furthermore, higher levels of autonomous motivation (Taylor et al., 2014), conscientiousness and openness (personality; Poropat, 2009; Zhang and Ziegler, 2016), BAS (temperament; Van Beek et al., 2013), positive affect and self-esteem (SWB; Quinn and Duckworth, 2007), self-perceived competence (Arefi et al., 2014), and intelligence and working memory (Roth et al., 2015; Peng and Fuchs, 2016) are expected to positively predict mathematical abilities. On the contrary, variables such as less O’s, lower levels of SES, lower parental aspirations, lower birth weight, a lower place in the birth order, and lower levels of emotional stability are expected to be associated with lower levels of mathematical performance. Also higher levels of controlled motivation, BIS (temperament) and negative affect (SWB) are supposed to result in lower math performances. Since this has never been explicitly investigated, no specific hypotheses are made for the different components (procedural calculation and fact retrieval) of mathematics.

## Materials and Methods

### Sample

This study was conducted on 114 children (79 females) from 3rd up until 6th grade in Flanders. There were 61 children in the MLD group and 53 children were recruited from the same classrooms to be a part of the control group. This was done to maximize the possibility that the O factors at school level (school learning environment) were the same in both groups. When recruiting someone from the same class was not possible (in 22.9% of the sample), a matched participant was selected based on age, grade, and gender.

All children in the MLD group met the criteria for MLD, and performed below average (substantially and quantifiably, below the 16th percentile), while performance was resistant to instruction (Ghesquière, 2014). Comorbidity with reading disabilities, Attention Deficit Hyperactivity Disorder (ADHD) and Developmental Coordination Disorder (DCD) was allowed, because of the high comorbidity rates with MLD (Scheiris and Desoete, 2008; Pieters et al., 2009, 2015; Kucian and von Aster, 2015). The mean intelligence (see section “Material”) was significantly lower in the MLD group (*M* = 91.119; *SD* = 1.508) compared to the control group (*M* = 103.359; *SD* = 1.614), *F*(1,115) = 30.725, *p* < 0.001. For SES, as measured by the Hollingshead Index (see section “Material”), there were no significant differences between groups, *F*(1,115) = 0.320, *p* = 0.573. Mean SES in the MLD group was 42.913 (*SD* = 10.512), and for the control group, the mean SES was 44.021 (*SD* = 10.577).

Children were recruited by spreading flyers, through social media, schools, psychologists, and language and speech therapists in Flanders. Children’s parents agreed for the research by signing an informed consent. This research was approved by the Ethical Committee of the Faculty of Psychology and Educational Sciences of Ghent University.

### Procedure

After parents agreed to the participation of their children, two appointments for the actual research were made. Each session lasted about 90 min while tests and questionnaires were administered individually for each child. For some participants, recent test data (max. 1.5 years) for intelligence and mathematics was already available from, for example, their psychologist. In that case, the available data (measured with the same tests as in this study) was used to prevent test–retest effects. Testing happened in a location chosen by the parents. Most often, this was the school or at home. The researcher gave standardized instructions and was available to answer questions. The first session started with the fact retrieval test. After that, intelligence and working memory were measured, followed by completing the questionnaires. The procedural accuracy math test was completed in the second session together with the remaining questionnaires. The specific order in which the questionnaires were filled out, could not be fully standardized, due to lot of individual differences between children regarding the duration of the standardized tests and their alertness during research. Therefore, the order was adapted to keep the child motivated to take part in the research by, for example, alternating longer with shorter questionnaires.

The questionnaires for the parents and the teacher were given to the parents during the first session, and handed back to the researchers after the research had finished.

### Material

Antecedent (A) and O factors were measured through questionnaires. More specifically, for the O factors, teachers were asked how many *years of experience* they had with teaching mathematics and how many hours of mathematical instructions the children received per week (*teaching hours*).

To measure A factors, parents were asked about their aspirations regarding the mathematical abilities of their children. They had to reflect on the score they wanted their child to have at the end of the current school year (in percentage). Additionally, information on *birth order* and *birth weight* of the child was collected. The SES of the family of the child was calculated using the Hollingshead index, combining the educational level and the current job of both parents into one score. The higher this score, the higher the SES of the family (Hollingshead, 1975, Unpublished). With regards to the P factors the following instruments were used.

#### Intelligence

It was measured using an abridged Dutch version of the Wechsler Intelligence Scale for Children-III (WISC-III-NL; Kort et al., 2005). The total intelligence quotient or IQ (*M* = 100; *SD* = 15) was obtained by combining the separate scores on the following subtests: Vocabulary, Similarities, Picture Concepts, and Block Design. The reliability of this short form was 0.92 and the distribution of total IQ-scores calculated with the short form did not significantly differ from the distribution of scores on the full intelligence test (Grégoire, 2000). Cronbach’s α of the total IQ in the current sample was 0.795.

#### Working Memory

It was assessed with the Working Memory Index of the Dutch version of the Clinical Evaluation of Language Fundamentals-4 (CELF-IV-NL; Kort et al., 2008). By combining the subtests of Forward and Backward Number Repetition and the subtest of Familiar Sequences, a score for working memory was calculated. Cronbach’s α was 0.786 for this sample.

#### Motivation for Mathematics

It was measured with the Dutch version of the Academic Self-Regulation Scale (Vansteenkiste et al., 2009) which consists of 24 questions which allow the calculation of the level of autonomous and controlled academic motivation. As suggested by the authors, the introduction for the questions was changed from “I am motivated to study because…,” to “I am motivated to study mathematics because …” in order to measure motivation with regards to mathematics specifically. The child had to respond on a 5-point Likert scale to statements such as “because I find this an important goal in my life” as an index of autonomous motivation and “because other people (e.g., parents, friends, teachers) oblige me to do so” to measure controlled motivation. The score for each scale was calculated by averaging the score on the items belonging to that scale. Cronbach’s α for this sample was 0.849 for autonomous and 0.727 for controlled motivation.

#### Personality

It was assessed by the Hierarchical Personality Inventory for Children (HiPIC; Mervielde and de Fruyt, 2009), filled out by the parents. This questionnaire was based on the Big Five Personality Theory (Costa and McCrae, 1992) and consisted of 144 items to measure the five personality traits: openness, conscientiousness, extraversion, agreeableness, and emotional stability (versus neuroticism). For each item, the parent had to indicate on a 5-point Likert scale how well that item applied to their child (e.g., “my child likes to learn new things”). The score for each personality trait was calculated using an algorithm in which some items were recoded inversely. The internal consistency of this questionnaire was good (α = 0.80–0.92) with a test–retest reliability of α = 0.72–0.83 (Egberink et al., 2010). Cronbach’s α for this sample was 0.868 for openness, 0.920 for conscientiousness, 0.642 for extraversion, 0.686 for agreeableness, and 0.905 for emotional stability.

#### Temperament

It was estimated with the Behavioral Inhibition (BIS) and Behavioral Activation (BAS) Questionnaire (Carver and White, 1994; translated by Franken et al., 2005). The children were asked to rate 24 items on a 4-point Likert scale. The score for BIS was calculated by averaging the score on seven items, for example, “I worry about making mistakes.” Two out of seven items were recoded reversely. For BAS, the score was calculated by averaging the score on 13 items, for example, “When I want something, I usually go all-out to get it.” The internal consistency of the scales have proven to be acceptable with BIS: α = 0.82 and BAS: α = 0.73 (Smits and Boeck, 2006). Cronbach’s α for this sample was 0.625 for BIS and 0.752 for BAS.

#### Subjective Well-Being

It was determined through the Dutch version of the Positive and Negative Affect Schedule (PANAS; Watson et al., 1988; translated by Engelen et al., 2006). Children indicated on a 5-point Likert scale how many negative (e.g., guilt and sadness) and positive (e.g., success and interest) emotions they experienced on a regular school day. Scores were calculated for the level of positive affect and the level of negative affect by averaging the score on 10 items. Cronbach’s α for this sample was 0.738 for positive affect and 0.708 for negative affect.

#### Self-Esteem

It was evaluated through the Dutch version of the Rosenberg self-esteem scale (Franck et al., 2008). Children had to judge 10 statements on a 4-point Likert scale. Examples of questions were: “In general, I am happy with myself” and “Sometimes, I feel like I am a failure.” A total self-esteem score was calculated by adding up the scores on all 10 items. Higher scores corresponded with higher levels of self-esteem whereas lower scores corresponded with lower levels of self-esteem. The internal consistency of the scale was high with a Cronbach’s α of 0.76 (De Corte et al., 2007). For this sample, α was 0.750.

#### Children’s Self-Perception of Academic Competence

It was assessed with the Self-Perception Profile for Children (Harter, 1985; translated by Veerman et al., 2004). This questionnaire measures how children perceive their own competences on several life domains. For the current study, self-perceived competence on the school level was used. The total score for that scale was calculated by adding up children’s scores on six questions. For each question, the child had to choose between two sentences and then indicate if that sentence is somewhat or entirely true for them. Every item received a score ranging from 1 to 4. The internal consistency was good, with a Cronbach’s α of 0.78 (Veerman et al., 2004). For the current sample, α for self-perceived academic competence was 0.809.

#### Mathematical Abilities

As outcome measures, fact retrieval speed and procedural accuracy were investigated. To measure the *fact retrieval speed*, the Arithmetic Number Fact Test (TTR; de Vos, 2002) was used. Children had to solve as much additions (e.g., “7 + 2”), subtractions (e.g., “6 - 5”), multiplications (e.g., “5 × 8”), divisions (e.g., “27 : 9”), or a mix of these exercises as possible in 5 min. The number of correct answers was used as outcome measure. This test has been standardized for Flanders on a sample of 10,059 children (Ghesquière and Ruijssenaars, 1994). The psychometric value of the test has been demonstrated with a Cronbach’s alpha of 0.90 (Desoete and Roeyers, 2005). For this sample Cronbach’s α was 0.954.

To measure *the procedural accuracy skills* of the child, the Cognitive Developmental skills in aRithmetics Test (CDR; Desoete and Roeyers, 2002) was administered. This test evaluates the understanding and proficiency needed to solve 90 exercises in a number-problem or word-problem format (e.g., “283 times more than -71 is …”; “27681 : 90 = …”; “Wim has 4.8 kg of flour. Jan has a double amount of flour. How many flour do Jan and Wim have together?”) without a time limit. The number of correct answers was calculated as outcome measure. The CDR has been standardized on 1332 Flemish children (Desoete and Roeyers, 2005). The internal consistency for this sample was Cronbach’s α = 0.860.

### Statistical Analyses

Before conducting statistical analyses to examine the hypotheses, the missing data (2.381% empty cells) was examined to asses if these items were missing completely at random (MCAR). Little’s MCAR test confirmed that data was missing completely at random, *χ*^2^(68, *n* = 114) = 63.569, *p* = 0.630. Missing values were imputed with the expectation-maximization technique.

Since the assumptions for parametric testing were met, Multivariate Analyses of Covariance (MANCOVA) were conducted on the A and P factors separately to examine the first hypothesis. Intelligence was used as covariate, since the MLD and control group significantly differed on IQ (see section “Sample”). To examine the second and third hypothesis, linear regression analyses were conducted with the A, O, and P factors as predictors for fact retrieval speed on the one hand and as predictors for procedural accuracy on the other hand. Interaction terms with group (MLD or control) were added for those variables of which the MANCOVA (Hypothesis 1) revealed that they differed between both groups. The raw scores of the mathematical tests were transformed into *z*-scores for each grade separately. This was done by standardizing them by the group means per grade, to correct for age effects. Finally, mediation analyses were conducted to test whether the effect of the A predictors on mathematical performance was mediated by the O and/or P predictors (Hypothesis 4).

## Results

### Hypothesis 1: There Will Be Differences in Antecedent and Propensity Indicators Between Children With and Without Mathematical Learning Disabilities (MLD)

A MANCOVA was conducted on the A predictors, with MLD status as independent variable and intelligence as covariate. Multivariate results revealed significant differences in A factors, *F*(4,112) = 21.738, *p* < 0.001, ${\text{η}}_{\text{p}}^{2}$ = 0.437. Furthermore, on the P factors, a similar MANCOVA was conducted.

Multivariate results revealed significant differences in P factors, *F*(12,104) = 7.760, *p* < 0.001, ${\text{η}}_{\text{p}}^{2}$ = 0.472. Univariate results, means (*M*) and standard deviations (*SDs*) for the A and P predictors can be found in **Tables 1**, **2**, respectively.

**Table 1:** Multivariate Analyses of Covariance (MANCOVA) on Antecedent predictors with intelligence as covariate.

	MLD		Control				
	*M*	*SD*	*M*	*SD*	*F*	*p*	${\text{η}}_{\text{p}}^{2}$
Parental Aspirations	63.923	8.769	82.668	8.909	86.352	<0.001***	0.429
SES	42.913	10.512	44.021	10.717	0.703	0.403	0.006
Birth Order	1.715	0.906	1.632	0.821	0.460	0.499	0.004
Birth Weight	3284.504	469.444	3288.083	421.967	0.024	0.877	0.000

*${\text{η}}_{\text{p}}^{2}$ interpretation: 0.020 = small effect; 0.130 = medium effect; 0.260 = large effect; ^∗^p < 0.050; ^∗∗^p ≤ 0.010; ^∗∗∗^p ≤ 0.001; MLD = Mathematical Learning Disabilities.*

**Table 2:** Multivariate Analyses of Covariance (MANCOVA) on Propensity predictors with intelligence as covariate.

	MLD		Control				
	*M*	*SD*	*M*	*SD*	*F*	*p*	${\text{η}}_{\text{p}}^{2}$
BIS	2.827	0.539	2.642	0.494	2.671	0.105	0.023
BAS	3.117	0.475	2.974	0.399	4.131	0.044*	0.035
Openness	85.095	11.067	94.780	10.244	8.459	0.004**	0.069
Conscientiousness	99.238	20.331	106.543	17.191	8.125	0.005**	0.066
Emotional Stability	45.175	11.007	51.854	10.352	4.501	0.036*	0.038
Autonomous Motivation	2.893	0.864	3.334	0.933	4.380	0.039*	0.037
Controlled Motivation	2.929	0.732	2.780	0.826	0.000	0.997	0.000
Working Memory	15.762	3.306	21.291	3.804	34.700	<0.001***	0.232
Positive Affect	3.544	0.625	3.606	0.623	0.118	0.731	0.001
Negative Affect	2.380	0.587	2.091	0.555	4.295	0.040*	0.036
Total Self-Esteem	19.714	4.567	22.200	3.638	5.392	0.022*	0.045
Self-Perceived Competence	12.762	3.609	18.324	3.644	43.636	<0.001***	0.275

*${\text{η}}_{\text{p}}^{2}$ interpretation: 0.020 = small effect; 0.130 = medium effect; 0.260 = large effect; ^∗^p < 0.050; ^∗∗^p ≤ 0.010; ^∗∗∗^p ≤ 0.001; MLD = Mathematical Learning Disabilities.*

Parents of children in the MLD group had significantly lower aspirations [antecedent (A)]. Additionally, these children scored significantly lower on openness, conscientiousness, emotional stability, autonomous motivation, self-esteem, self-perceived competence, intelligence, and working memory when compared to children in the control group. In contrast, they scored significantly higher for negative affect and BAS (P).

### Hypothesis 2: A Selection of Antecedent, Opportunity, and Propensity Indicators Will Predict Fact Retrieval Speed

Multiple regression analysis with the A variables, group (MLD or control) and interaction of parental aspirations × group as predictors for the *z*-scores on the TTR revealed a significant regression equation, *F*(6,114) = 17.256, *p* < 0.001, *R*^2^= 0.483. The regression coefficients, standard deviations and the significance tests for the different predictors can be found in **Table 3A**.

**Table 3:** Multivariate Regression Models with Antecedent, Opportunity, or Propensity predictors on fact retrieval speed (tested with the TTR).

		B	*SE*	β	*t*	*p*
**(A) Antecedent predictors**	(constant)	1.548	0.917		1.687	0.094
*F* = 17.256, *R*^2^ = 0.483 (*p* < 0.001^∗∗∗^)	Parental Aspirations	-0.024	0.011	-0.313	-2.215	0.029*
	SES	-0.001	0.006	-0.006	-0.080	0.936
	Birth Order	-0.123	0.080	-0.109	-1.539	0.127
	Birth Weight	-9.849^e-005^	0.000	-0.045	-0.648	0.518
	Group (MLD, Control)	-5.450	1.184	-2.790	-4.603	<0.001***
	Group × Parental Aspirations	0.085	0.016	3.623	5.357	<0.001***
**(B) Opportunity predictors**	(constant)	-0.820	0.358		-2.292	0.024
*F* = 4.079, *R*^2^ = 0.066 (*p* = 0.019^∗^)	Teachers’ Experience (years)	0.020	0.010	0.185	2.019	0.046*
	Hours of math per week	0.108	0.066	0.149	1.622	0.108
**(C) Propensity predictors**	(constant)	1.271	1.686		0.754	0.453
*F* = 4.281, *R*^2^ = 0.525 (*p* < 0.001^∗∗∗^)	BIS	-0.072	0.163	-0.039	-0.444	0.658
	BAS	0.181	0.254	0.082	0.715	0.477
	Openness	0.016	0.011	0.196	1.443	0.155
	Conscientiousness	-0.009	0.006	-0.173	-1.393	0.167
	Emotional Stability	-0.007	0.012	-0.075	-0.541	0.590
	Autonomous Motivation	0.006	0.130	0.005	0.043	0.966
	Controlled Motivation	0.094	0.103	0.075	0.919	0.360
	Working Memory	0.035	0.050	0.396	0.702	0.484
	Intelligence	-0.030	0.011	-0.405	-2.672	0.009**
	Positive Affect	-0.168	0.146	-0.107	-1.157	0.250
	Negative Affect	-0.092	0.195	-0.056	-0.474	0.637
	Total Self-Esteem	0.041	0.026	0.180	1.549	0.125
	Self-Perceived Competence	-0.040	0.034	-0.188	-1.178	0.242
	Group (MLD, Control)	-4.519	2.480	-2.313	-1.822	0.072
	Group × BAS	0.176	0.386	0.272	0.455	0.650
	Group × Openness	-0.020	0.018	-0.986	-1.136	0.259
	Group × Conscientiousness	0.013	0.010	0.742	1.343	0.182
	Group × Emotional Stability	0.011	0.017	0.311	0.663	0.509
	Group × Autonomous Motivation	-0.056	0.175	-0.101	-0.318	0.751
	Group × Working Memory	0.035	0.050	0.396	0.702	0.484
	Group × Intelligence	0.029	0.016	1.584	1.897	0.061
	Group × Negative Affect	0.002	0.296	0.002	0.006	0.995
	Group × Total Self-Esteem	-0.063	0.044	-0.729	-1.409	0.162
	Group × Self-Perceived Competence	0.166	0.050	1.611	3.317	0.001**

*^∗^p < 0.050; ^∗∗^p ≤ 0.010; ^∗∗∗^p ≤ 0.001; MLD = Mathematical Learning Disabilities.*

**FIGURE 3:**
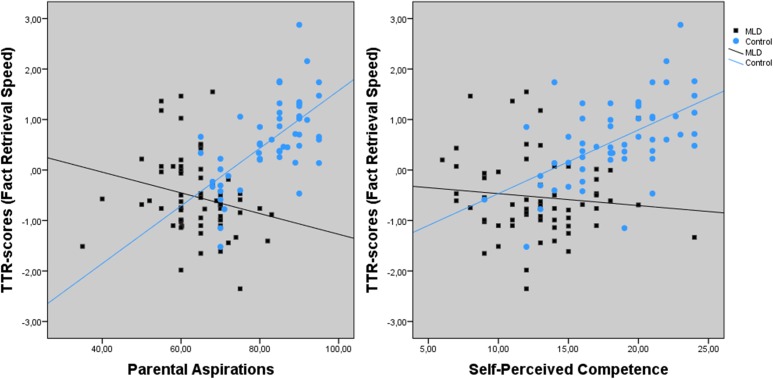
Interaction effect between Parental Aspirations, Self-Perceived Competence, and group (MLD or control) for fact retrieval speed.

**Table 3A** demonstrates a significant main effect for parental aspirations and group (MLD or control) on fact retrieval scores. The interaction effect of parental aspirations × group was also significant (see left part of **Figure 3**). The regression line for the MLD group was non-significant, *F*(1,60) = 3.290, *p* = 0.075, *R*^2^ = 0.051. However, parental aspirations were predictive for fact retrieval speed in the control group, *F*(1,52) = 33.163, *p* < 0.001, *R*^2^ = 0.385.

Next, the multivariate regression with the O predictors for fact retrieval was significant, *F*(2,114) = 4.079, *p* < 0.001, *R*^2^= 0.066. The univariate results and coefficients can be found in **Table 3B** and indicated that the years of experience the teacher had was predictive for fact retrieval speed.

Further, the multiple regression analysis with the P variables, group (MLD or control) and separate interaction variables (Group × BAS, × openness, × conscientiousness, × emotional stability, × autonomous motivation, × self-esteem, × negative affect, × self-perceived competence, × intelligence, and × working memory) as predictors was conducted on fact retrieval speed. This analysis revealed a significant regression equation, *F*(24,114) = 4.281, *p* < 0.001, *R*^2^= 0.525 (see **Table 3C**).

Results showed a significant main effect of intelligence and a trend for group on fact retrieval speed. Furthermore, the interaction of self-perceived competence × group was significant (see right part of **Figure 3**). The regression lines per group indicated that self-perceived competence was a significant predictor for fact retrieval speed in the control group, *F*(1,52) = 24.295, *p* < 0.001, *R*^2^ = 0.314, but not in the MLD group *F*(1,60) = 0.711, *p* = 0.402, *R*^2^ = 0.012.

### Hypothesis 3: A Selection of Antecedent, Opportunity, and Propensity Indicators Will Predict Procedural Accuracy

Linear regression analyses were conducted for the third hypothesis to predict the *z*-scores on the CDR. The same interaction variables as in Hypothesis 2 were added into the model.

The multiple regression analysis with the A variables, group (MLD or control) and interaction of parental aspirations × group as predictors for procedural accuracy revealed a significant regression equation, *F*(6,114) = 19.819, *p* < 0.001, *R*^2^= 0.517 (see **Table 4A**).

**Table 4:** Multivariate Regression Models with Antecedent, Opportunity, or Propensity predictors on procedural accuracy (tested with the CDR).

		B	*SE*	β	*t*	*p*
**(A) Antecedent predictors**	(constant)	-2.133	0.896		-2.358	0.020
*F* = 19.819, *R*^2^ = 0.517 (*p* < 0.001^∗∗∗^)	Parental Aspirations	0.017	0.011	0.227	1.659	0.100
	SES	0.014	0.006	0.146	2.171	0.032*
	Birth Order	0.080	0.078	0.070	1.027	0.307
	Birth Weight	0.000	0.000	-0.046	-0.686	0.494
	Group	-0.486	1.156	-0.246	-0.420	0.675
	Group × Parental Aspirations	0.017	0.015	0.735	1.125	0.263
**(B) Opportunity predictors**	(constant)	-0.812	0.362		-2.243	0.027
*F* = 3.898, *R*^2^ = 0.063 (*p* = 0.023^∗^)	Teachers’ Experience (years)	0.019	0.010	0.180	1.965	0.052
	Hours of math per week	0.107	0.067	0.146	1.596	0.113
**(C) Propensity predictors**	(constant)	-3.834	1.369		-2.800	0.006
*F* = 8.770 *R*^2^ = 0.694 (*p* < 0.001^∗∗∗^)	BIS	-0.029	0.132	-0.016	-0.223	0.824
	BAS	0.203	0.206	0.091	0.983	0.328
	Openness	0.000	0.009	0.002	0.018	0.986
	Conscientiousness	-0.013	0.008	-0.720	-1.623	0.108
	Emotional Stability	-0.020	0.014	-0.548	-1.453	0.150
	Autonomous Motivation	0.034	0.105	0.031	0.319	0.750
	Controlled Motivation	-0.045	0.083	-0.035	-0.534	0.594
	Working Memory	-0.002	0.028	-0.008	-0.065	0.949
	Intelligence	0.023	0.009	0.317	2.608	0.011*
	Positive Affect	-0.323	0.118	-0.203	-2.731	0.008**
	Negative Affect	0.302	0.158	0.179	1.904	0.060
	Total Self-Esteem	-0.031	0.021	-0.134	-1.434	0.155
	Self-Perceived Competence	-0.015	0.028	-0.068	-0.533	0.595
	Group (MLD, Control)	-0.645	2.014	-0.326	-0.320	0.750
	Group × BAS	-0.105	0.313	-0.160	-0.334	0.739
	Group × Openness	0.014	0.014	0.694	0.995	0.322
	Group × Conscientiousness	-0.013	0.008	-0.720	-1.623	0.108
	Group × Emotional Stability	-0.020	0.014	-0.548	-1.453	0.150
	Group × Autonomous Motivation	-0.223	0.142	-0.402	-1.568	0.120
	Group × Working Memory	0.082	0.041	0.908	2.006	0.048*
	Group × Intelligence	-0.017	0.013	-0.886	-1.322	0.190
	Group × Negative Affect	-0.038	0.240	-0.043	-0.159	0.874
	Group × Total Self-Esteem	0.071	0.036	0.819	1.973	0.052
	Group × Self-Perceived Competence	0.128	0.041	1.225	3.141	0.002**

*^∗^p < 0.050; ^∗∗^p ≤ 0.010; ^∗∗∗^p ≤ 0.001; MLD = Mathematical Learning Disabilities.*

There was a significant main effect of SES on procedural accuracy and a trend towards significance for parental aspirations as predictor. Next, the multivariate regression with the O factors as predictors for procedural accuracy was significant, *F*(2,114) = 3.898, *p* < 0.001, *R*^2^= 0.063. The univariate results and coefficients can be found in **Table 4B**. None of the predictors seemed to be predictive on the univariate level, however, the years of experience of the teacher was marginally significant.

A multiple regression analysis with the P variables, group (MLD or control) and separate interaction variables (Group × BAS, × openness, × conscientiousness, × emotional stability, × autonomous motivation, × self-esteem, × negative affect, × self-perceived competence, × intelligence, and × working memory) as predictors was conducted on procedural accuracy. This analysis revealed a significant regression equation, *F*(24,114) = 8.770, *p* < 0.001, *R*^2^= 0.694 (see **Table 4C**).

**FIGURE 4:**
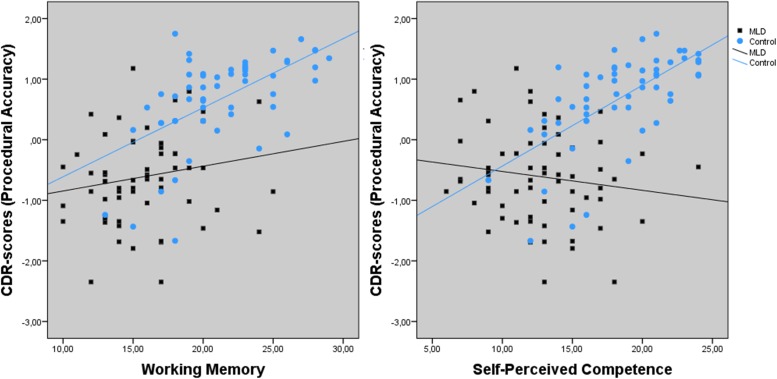
Interaction effect between Working Memory, Self-Perceived Competence, and group (MLD or control) for procedural accuracy.

The univariate results indicated a significant main effect for positive affect and intelligence on procedural accuracy. Furthermore, there was a trend towards a significant main effect for negative affect, emotional stability, and conscientiousness. The interaction effects for group × working memory and group × self-perceived competence were significant (see **Figure 4**). Working memory was a significant predictor for procedural accuracy in the control group, *F*(1,52) = 26.117, *p* < 0.001, *R*^2^ = 0.330, but not in the MLD group *F*(1,60) = 2.025, *p* = 0.160, *R*^2^ = 0.032. Also for self-perceived competence, a significant regression equation was found in the control group, *F*(1,54) = 37.647, *p* < 0.001, *R*^2^ = 0.415, but not in the MLD group, *F*(1,62) = 1.380, *p* = 0.245, *R*^2^ = 0.022. There was a trend towards a significant effect for the interaction between group × self-esteem on procedural accuracy.

### Hypothesis 4: The Predictive Value of Some Antecedent Variables Will Be Mediated Through Some Opportunity and Propensity Variables

Mediation analyses were conducted (Hypotheses 2 and 3) in “Process” by Andrew Hayes (Field, 2016). Since parental aspirations × group (MLD or control) was a significant predictor for fact retrieval speed, it was examined if this effect was mediated through teachers’ experience (significant O predictor) on the one hand and through self-perceived competence × group and intelligence (significant P predictors) on the other hand.

The results revealed that the effect of parental aspirations on fact retrieval speed was not mediated through teachers’ experience for both the MLD and the control group. Results for the MLD group are *b* = 0.000, BCa CI [-0.007, 0.004] and for the control group *b* = 0.000, BCa CI [-0.007, 0.008].

Further, a significant indirect effect of parental aspirations on fact retrieval speed through self-perceived competence was revealed for both the MLD group, *b* = 0.004, BCa CI [0.000, 0.011], and the control group, *b* = 0.013, BCa CI [0.002, 0.028]. The indirect effect of parental aspirations through intelligence was non-significant, *b* = 0.004, BCa CI [-0.002, 0.012].

Because SES was a significant predictor for procedural accuracy, a possible mediation through intelligence and positive affect on the one hand and self-perceived competence × group (significant P predictors) on the other hand was examined. Mediation through intelligence was significant, *b* = 0.014, BCa CI [0.005, 0.024]. No indirect effect was found for SES on procedural accuracy through positive affect, *b* = -0.002, BCa CI [-0.006, 0.001]. The indirect effect of SES on procedural accuracy through self-perceived competence was non-significant for both the MLD group, *b* = -0.000, BCa CI [-0.007, 0.007] and the control group, *b* = 0.005, BCa CI [-0.001, 0.014].

## Discussion

Throughout the last decade, several predictors of mathematical learning have been proposed. To evaluate individual differences and the unique contribution of predictors, it is important to take into account the interrelationships between those predictors. Within the O-P Model, it is suggested that learning occurs as the result of A, O, and P factors (Byrnes and Miller, 2007). Studies on large secondary datasets have revealed the value of this model in kindergarten (Byrnes and Wasik, 2009; Wang and Byrnes, 2013), the beginning of primary school (Byrnes and Wasik, 2009), and in secondary school (Byrnes and Miller, 2007, 2016). To the best of our knowledge, no studies have examined this model by collecting primary data in the second half of primary school in a group of children with and without MLD. This study aimed to fill this gap in the existing research by investigating whether children with and without MLD differ on A and P variables and assess whether information on the combination of these variables adds to the current knowledge on mathematical abilities and disabilities. Moreover as mathematics has been described as componential in nature (Dowker, 2015), the relationship with both fact retrieval speed and procedural calculation is examined and compared.

### Differences in Antecedent and Propensity Indicators Between Children With and Without MLD

Results showed significant differences in both A and P factors when comparing children with and without MLD.

In contrast with the hypotheses, children with MLD did not differ significantly from typically developing children on the A factors, with regards to birth weight, SES, and birth order. However, they did differ on parental aspirations. Parents had significantly lower aspirations toward mathematical learning when their children had MLD. A large effect size was found. Further research is needed to examine whether these lower aspirations were caused by children’s continuous struggle with math learning or if the lower math performances of children with MLD are due to lower parental aspirations.

With regards to the P factors, children with and without MLD differed on temperament, personality, motivation, working memory, SWB, self-esteem, and self-perceived competence, after controlling for intelligence. Regarding temperament, children with MLD had higher scores on BAS compared to typically developing children. However, in contrast with Van Beek et al. (2013), results did not show significant differences between both groups for BIS. This unexpected result could be due to a power problem. Our findings seem to indicate that children with MLD might be more sensitive for rewards than peers without MLD. This might implicate that teachers should use rewards and positive consequences as a lever to enhance their mathematical performances.

Concerning personality, children with MLD were less open to new experiences, were less conscientious, and had lower scores for emotional stability compared to peers in the control group. The effects of openness and conscientiousness were larger than the effect of emotional stability (versus neuroticism). These results are in line with earlier research which indicated openness and conscientiousness as the personality traits most associated with mathematical performance (Poropat, 2009; Zhang and Ziegler, 2016).

Analysis of the P factor of motivation indicated no differences in the amount of controlled motivation (where the force to fulfill a task is external; e.g., a reward) between children with and without MLD. However, children with MLD had lower levels of autonomous motivation (where one fulfills a task for an internal reason such as passion or future relevance of the topic) when compared to controls. This indicates that children from both groups were equally motivated for mathematics because they had to, whereas children with MLD were less motivated for mathematics because they wanted to. Next, in line with literature (Roth et al., 2015; Peng and Fuchs, 2016), results revealed that children with MLD experienced more difficulties with working memory when compared to typically developing children. This effect was between medium and large indicating that working memory problems might be impactful for children with MLD.

Furthermore, children with MLD experienced more negative affect on a regular school day than their typically developing peers in the same school context. There were no significant group differences found for positive affect. When examining self-esteem, data revealed that children with MLD reported lower self-esteem than their peers without MLD. These results indicate the impact of MLD on the SWB of children. Even though they seemed to experience the same amount of positive feelings as their typically developing peers, they experienced more negative affect and more negative feelings toward themselves. In line with the reciprocal-effects model (Guay et al., 2003; Seaton et al., 2015), it is possible that having MLD impacts children’s SWB, which in its turn affects their mathematical abilities resulting in more severe math problems.

Finally, children with MLD perceived their own academic competences much lower (large effect size) than did typically developing children, which indicated that they were aware of their own lower capacity in mathematics.

### The Predictive Value of Antecedent, Opportunity, and Propensity Factors for Math Performance

First, some A, O, and P factors were predictive for fact retrieval speed. The combination of SES, birth weight, parental aspirations, and birth order as A predictors explained 48.3% of variance in fact retrieval speed. In line with earlier research (Blevins-Knabe et al., 2007; Kleemans et al., 2012; Niklas et al., 2016), parental aspirations towards mathematical performance were a significant A predictor. Parents who wanted their children to score higher at the end of the current school year tended to have children who performed better in mathematics. Nonetheless, in our dataset, parental aspirations were important predictors only for typically developing children. Additionally, this effect was partially mediated through children’s self-perceived competence. However, based on the current study, no conclusions can be drawn about the direction of the effect. For instance, it is possible that lower math abilities of children influenced parental aspirations and children’s self-perceived competence. In contrast, it is possible that lower parental aspirations influenced children’s self-perceived competence and in their turn resulted in lower math abilities. However, reciprocal effects are also a possibility. Additional and longitudinal studies are necessary to understand the effect of parental aspirations more clearly. Moreover, not finding a predictive effect of parental aspirations for fact retrieval speed in the MLD group could be associated with severity or specificity of MLD as a developmental disorder. It is possible that persevering fact retrieval or fluency problems that characterize MLD cannot be influenced by parents’ expectations.

In contrast with the available literature on A predictors, SES, birth weight, and birth order did not significantly predict fact retrieval speed. The lack of association between SES and fact retrieval speed might be explained by the limited sample size or by the nature of fact retrieval mathematics as component of mathematics. Since retrieving arithmetic facts depends on drill and memorization, it could be less susceptible to the job and educational level of parents than other components of mathematics. On birth weight, the literature focused especially on effects of extremely low birth weight (<1500 g; De Rodrigues et al., 2006; Chatterji et al., 2014). In the current sample, none of the children had birth weights below 2000 g. Further, the results of this study did not confirm earlier studies which reported better performance in academic contexts when higher in the birth order (Hotz and Pantano, 2015). This might be due to the small variability in birth order places of the participants, since 86.4% of this sample was the first or second born child in their family.

This study confirmed that mathematical abilities improve with more O’s (Zhang, 2008; Byrnes and Wasik, 2009). The O’s explained 6.6% of the variance in fact retrieval speed. More experienced teachers seem to have a positive impact on children’s math performances. The number of hours of mathematics instruction children received per week had no significant effect. This might indicate that not the quantity (number of hours) but the quality (teachers’ experience) of instruction matters. Furthermore, mediation of A through O variables was not found in the current study since parental aspirations did not predict teachers’ experience. This might be explained by the specific selection of O variables in the current study compared to earlier studies on the O-P Model (e.g., Byrnes and Wasik, 2009) which included richer O measurements. Future research should measure O factors more broadly, whereas now teachers were only asked about their years of experience and how many hours they taught mathematics.

The P variables included in this study explained 52.5% of the variance found in fact retrieval speed, which indicated that the P factors are the strongest predictors for retrieving arithmetic facts. This is in line with earlier research on the O-P Model (Byrnes and Wasik, 2009). Both intelligence and self-perceived competence were significant predictors in earlier studies (Arefi et al., 2014; Peng and Fuchs, 2016). However, the effect of self-perceived competence was only present in typically developing children, but not in children with MLD. Analog to parental aspirations, this might be related to the severity of fact retrieval deficits and might not be influenced by having higher perceptions of your own competences. It is important to note that also here, no conclusions can be drawn about the direction of the effects. Longitudinal studies are necessary but in line with the literature we can expect reciprocal effects between academic self-concept and academic achievement (Guay et al., 2003; Seaton et al., 2015).

Second, procedural accuracy could be predicted by some of the A, O, and P factors. The combination of SES, birth weight, parental aspirations, and birth order as A predictors explained 51.7% of the variance in procedural accuracy. Children with higher SES, performed better in procedural calculation, which is in line with earlier research (Wang et al., 2013). The data on parental aspirations of this study did not confirm its predictive value for procedural calculation, in contrast with the existing literature (Blevins-Knabe et al., 2007; Kleemans et al., 2012; Niklas et al., 2016). However, this could be related to power-issues since there was a trend towards a significant effect.

Analysis of procedural calculation, confirmed that mathematical abilities become better with more O’s (Zhang, 2008; Byrnes and Wasik, 2009). O’s explained 6.3% of the variance. There was a trend towards a significant association for the years of experience the teacher had. The same conclusions could be drawn as for fact retrieval fluency.

The P variables were the most predictive for procedural calculation, which is in line with earlier research on the O-P Model (Byrnes and Wasik, 2009). They explained 69.4% of variance. Intelligence, positive affect, working memory, and self-perceived competence were significant predictors. Higher levels of intelligence were associated with higher scores in procedural accuracy, which is in line with the literature (Roth et al., 2015; Peng and Fuchs, 2016). Moreover, the effect of SES on procedural calculation abilities was partially mediated through intelligence in this sample. With regards to working memory capacity, a significant association with procedural accuracy was found in the typically developing children. This association is in line with work of De Weerdt et al. (2013). A positive association between self-perceived competence, and procedural calculation was found, confirming results from earlier studies (Arefi et al., 2014). However, the effect of self-perceived competence was only present in typically developing children, not in children with MLD. This is analog to the findings on self-perceived competence and fact retrieval speed. Again, it is reasonable to expect reciprocal effects between self-perceived competence and procedural accuracy (Guay et al., 2003; Seaton et al., 2015). Not finding an effect of self-perceived competence for children with MLD might be the result of severe deficits that are not susceptible for influences of self-perceived competence. Additionally, a negative association was found between positive affect and procedural accuracy, which is in contrast with the literature on SWB (Quinn and Duckworth, 2007). Additional research is necessary to confirm and explain this finding.

Third, when comparing the effects of A, O and, P variables on fact retrieval speed with procedural accuracy, some important similarities and differences should be noted. Antecedent (A) factors explained about half of the variance in both types of math learning. However, for fact retrieval, the most important predictor was parental aspirations, whereas for procedural accuracy, SES seemed more important than parental aspirations. Furthermore, results revealed that the impact of A variables was mediated through P variables. More specifically, the effect of parental aspirations on fact retrieval speed was partially mediated through children’s self-perceived competence and the effect of SES on procedural accuracy was partially mediated through intelligence. These results provide evidence for the structure of the O-P Model (Byrnes and Miller, 2007). Nonetheless, in contrast with the proposed structure of the model, the data of this study did not confirm the mediation of A variables through O factors, which could be explained by the rather limited measures of O variables in the current study. Future research should include richer measurements of O’s. For O variables, about 6% of the variance for each of the mathematical components could be explained. Teachers’ years of experience proved to be an important factor, which highlights the importance of the quality and not quantity of instruction. The P variables were the strongest predictors of math abilities and more variance could be explained for procedural accuracy (about 70%) compared to fact retrieval fluency (about 50%). In earlier research on the O-P Model (Byrnes and Miller, 2007), P variables were also the strongest predictors for outcome. However, in the current study different P’s seemed to be predictive for fact retrieval compared to procedural calculation. Intelligence and self-perceived competence contributed to both types of mathematics, whereas positive affect and working memory were only predictive for procedural calculation. This could be related to the nature of the tasks used. In procedural calculation, children have to understand the mathematical principles and procedures to find the correct answer. Compared to fact retrieval tasks where arithmetic facts have to be memorized and retrieved, it makes sense that procedural accuracy is more susceptible to other influences than intelligence (e.g., positive affect and working memory). Moreover, fact retrieval depends on drill and memorization and therefore retrieving arithmetical facts might be less susceptible to the influence of P variables in general. This could also be the explanation of why more variance is explained by P variables for procedural calculation compared to fact retrieval fluency.

Finally, in contrast with the hypotheses, no association with mathematical abilities was found for motivation, personality and temperament. Although the literature on personality describes conscientiousness and openness as the most predictive personality traits for academic performances (Poropat, 2009; Zhang and Ziegler, 2016), when these variables were simultaneously investigated with other P variables in a holistic model, no predictive value was revealed. This emphasizes the importance of taking into account the interrelationship between several predictors in order to thoroughly understand mathematical development. However, we did find significant differences in personality when comparing children with and without MLD (see section “Differences in Antecedent and Propensity Indicators Between Children With and Without MLD”). Regarding autonomous motivation and temperament factors (BIS and BAS), this study did not reveal a predictive value for mathematical abilities when investigated within a holistic model. This is in contrast with the existing literature on motivation (Taylor et al., 2014) and temperament. Nonetheless, we did find significant differences for these variables when comparing children in the MLD group with their typically developing peers (see section “Differences in Antecedent and Propensity Indicators Between Children With and Without MLD”). When trying to predict outcome, it seems to be important to examine multiple variables within a holistic framework and to compare children with and without MLD.

### Limitations and Suggestions for Future Research

Every study has limitations. In this study, the sample size was rather small which could have repercussions on results. The sample size in previous work on the O-P Model was much larger. However, the data used in this study were primary collected data from an MLD population, whereas all previous studies used secondary data from a general population. We should take into account that some significant associations or differences on population level could not be detected within this sample due to power issues but it is a strength that data is collected within a clinical population. Future research should collect primary data on larger sample sizes.

Furthermore, in previous studies on the O-P Model, prior knowledge was a strong predictor of math performance (Byrnes and Miller, 2007). In the current study, this variable could not be examined since we were not able to collect data that was comparable across children. The children lived in different cities and attended different schools. Additionally, there were no standardized measures of prior knowledge previously administered in all children. However, in a follow-up study the collected measures of current skills will be used as prior knowledge for their skills in wave 2.

Finally, because this was a cross-sectional study, no conclusions about cause-and-effect can be made. Additional, longitudinal studies are currently being conducted.

## Conclusion and Implications for Practice

Despite the limitations, our results support the fact that children with MLD differ on A (e.g., parental aspirations) as well as on several (both cognitive and non-cognitive) P indicators. An exclusive P approach or only assessing cognitive predictors might not be a good idea.

Second, the O-P Model revealed to be applicable to the study of children with MLD. However, our findings also demonstrated that general protocols for the assessment of procedural calculation abilities or fact retrieval speed should not be implemented in the same way to test children with MLD and their typically developing peers. Since different predictors for mathematical abilities were found in children with and without MLD and in line with the componential nature of mathematics, adequately customized and broad assessments remain needed. Regarding procedural calculation, our findings revealed the importance of questionnaires on SES and tests on intelligence, positive affect, working memory, self-esteem, and self-perceived competence. With regards to fact retrieval speed, questionnaires on parental aspirations, and teachers’ experience, intelligence tests and a questionnaire on self-perceived competence seem indicated.

Finally, the current findings seem to indicate that children with MLD might be more sensitive to rewards, less open to new experiences and less conscientious. In addition, they were less autonomously motivated and had lower levels of SWB, lower self-esteem and lower self-perceived competence. These findings suggest the importance of positive feedback and psychoeducation including the enhancement of the autonomous motivation for mathematics in those children, in addition to the focus on their math acquisition. Therapy should focus on their strengths and reward small positive steps in the correct direction.

## Ethics Statement

This study was carried out in accordance with the recommendations of “The Ethical Committee of the Faculty of Psychology and Educational Sciences of Ghent University” with written informed consent from the parents of all subjects. All parents gave written informed consent in accordance with the Declaration of Helsinki.

## Author Contributions

EB performed the data collection, data analysis, and writing of the manuscript. AD supervised the data analysis and wrote the manuscript.

## Conflict of Interest Statement

The authors declare that the research was conducted in the absence of any commercial or financial relationships that could be construed as a potential conflict of interest.
